# Uncleaved signal peptide drives Golgi-bypassing secretion of the baculovirus fusion protein GP64

**DOI:** 10.1128/spectrum.03445-25

**Published:** 2026-07-24

**Authors:** Dingxiang Zhu, Yufeng Hao, Jingsong Zhang, Ying Xu, Bifang Hao, Jinshan Huang

**Affiliations:** 1 Jiangsu Key Laboratory of Sericultural and Animal Biotechnology, School of Biotechnology, Jiangsu University of Science and Technology 12676, Zhenjiang, China; 2 Key Laboratory of Silkworm and Mulberry Genetic Improvement, Ministry of Agriculture and Rural Affairs, Sericultural Scientific Research Center, Chinese Academy of Agricultural Sciences 625370, Zhenjiang, China; Oklahoma State University College of Veterinary Medicine, Stillwater, Oklahoma, USA

**Keywords:** baculovirus, membrane proteins, protein secretion, signal peptide, BmNPV

## LETTER

Viral membrane fusion proteins (MFPs) must be correctly folded, oligomerized, and trafficked to the plasma membrane (PM) to enable infection (1). In most enveloped viruses, these processes occur through the canonical ER–Golgi secretory pathway, where glycoproteins undergo folding, post-translational modifications, and assembly (2). Although this paradigm is well established, whether viral fusion proteins can reach the cell surface through alternative routes remains largely unexplored

GP64 is the essential MFP mediating budded virions (BVs*)* infection in Group I alphabaculoviruses. Unlike most baculoviruses, Bombyx mori nucleopolyhedrovirus (BmNPV) is a severe pathogen of the domesticated silkworm, causing significant economic loss. We recently demonstrated that BmNPV GP64 retains its signal peptide (SP) in *B. mori*-derived (BmN) cells but not in nonhost Sf9 cells, and that this uncleaved SP is essential for GP64 localization to the PM, determining BmNPV’s dual budding and fusion mechanisms (3–5). The trafficking pathway underlying GP64 secretion, however, has remained unclear.

**Fig 1:**
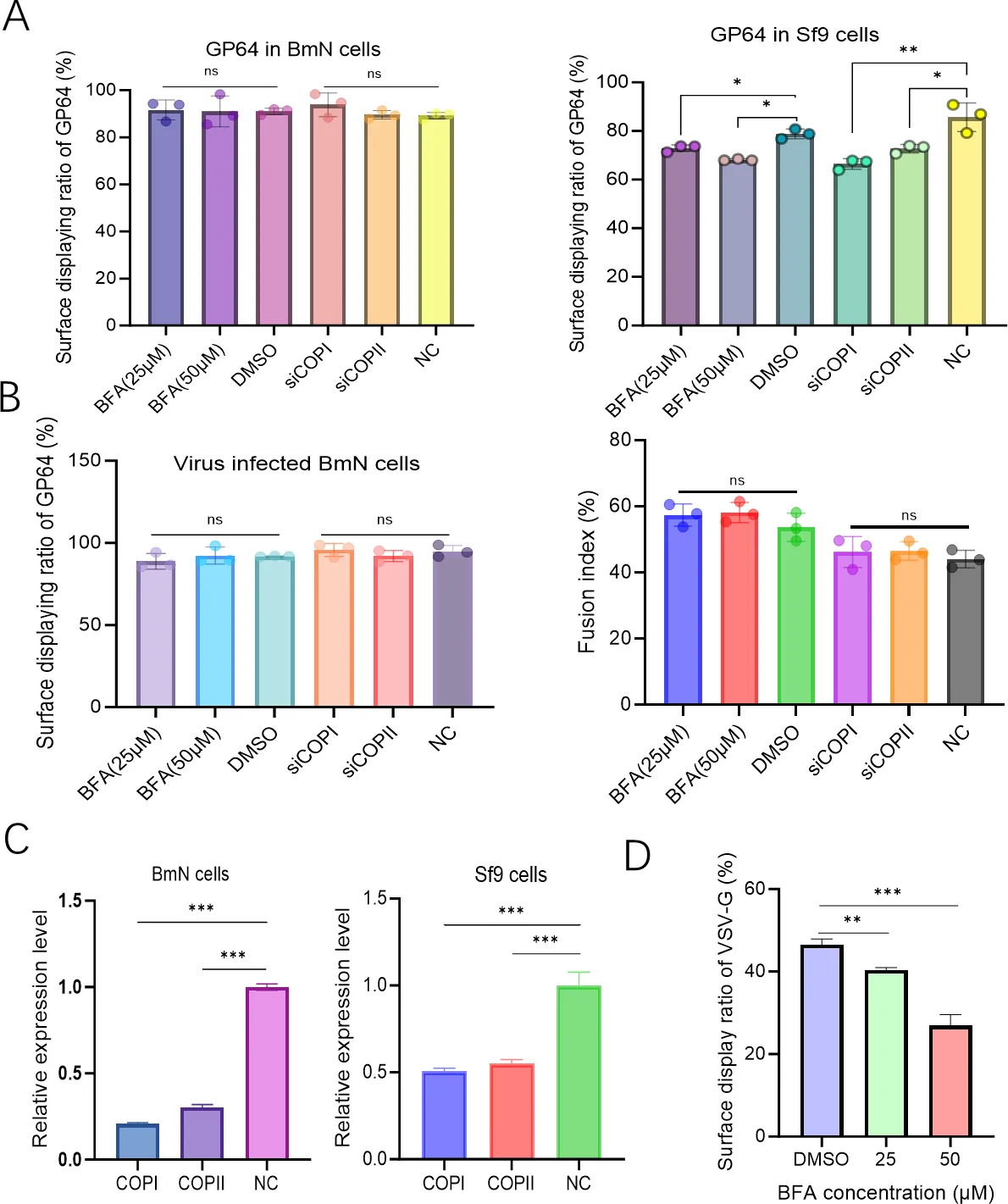
GP64 trafficking is resistant to ER–Golgi disruption in BmN cells but not in Sf9 cells. (**A**) Cell-based ELISA (cELISA) analysis of GP64 surface expression in BmN and Sf9 cells transiently expressing GP64. Cells were transfected with pIZ/V5-gp64 and fixed at 72 h post transfection (h p.t.), followed by either permeabilization with Triton X-100 or no permeabilization before cELISA analysis as described (6). The surface-display ratio was calculated as the absorbance of non-permeabilized cells (surface expression) divided by that of permeabilized cells (total expression). Data are presented as mean ± SD (*n* = 3). (**B**) Effects of ER–Golgi disruption on GP64 surface localization and fusogenic activity in BmNPV-infected BmN cells. Cells were infected with BmNPV (MOI = 3) for 2 h and then treated with BFA for 4 h or subjected to siRNA-mediated depletion of COP complex subunits as described (4). At 48 h post-infection, GP64 surface expression was quantified by cELISA (left panel), and low-pH (pH 4.5)-induced cell–cell fusion was assessed (right panel). The fusion index was calculated as the percentage of fused nuclei relative to total nuclei from ten randomly selected fields as described (6). (**C**) Analysis of siRNA interference efficiency of COPI/II by qPCR in BmN and Sf9 cells. BmN and Sf9 cells were transfected with siRNA, and total RNA was extracted using TRIzol reagent, as previously described (4). GAPDH served as the internal control. Relative gene expression was calculated by the 2^−ΔΔC*t*^ method, with data derived from three biological replicates and three technical replicates. (**D**) Golgi-dependent trafficking of vesicular stomatitis virus glycoprotein G (VSV-G) in BmN cells. BmN cells were transfected with pIZ/V5-VSV-G for 6 h, followed by BFA treatment for 6 h. At 48 h p.t., cells were subjected to cELISA using a VSV-G rabbit monoclonal antibody (Cell Signaling), with or without prior permeabilization with Triton X-100. Data are presented as mean ± SD (*n* = 3). Statistical significance is indicated (*P < 0.05, **P < 0.01, ***P < 0.001; ns, not significant).

To determine whether BmNPV GP64 secretion depends on Golgi-mediated transport, GP64 was transiently expressed in BmN and Sf9 cells maintained with TC100 medium supplemented with 10% fetal bovine serum and in Sf-900 II SFM, respectively, as described (5). ER-Golgi trafficking was then disrupted either by brefeldin A (BFA) treatment or siRNA-mediated knockdown of COP complex subunits targeting *Bombyx mori* and *Spodoptera frugiperda* (Table 1). The surface display ratio of GP64 secretion was calculated as the absorbance of non-permeabilized cells (surface expression) divided by that of permeabilized cells (total expression) by cell-based ELISA (cELISA) using anti-GP64 antibodies as described (4). Strikingly, blocking ER–Golgi transport had no significant effect on GP64 secretion in BmN cells but caused a marked reduction in Sf9 cells (Fig. 1A), suggesting Golgi-bypassing secretion in the host context. The same pattern held in infection experiments: in BmNPV-infected BmN cells (multiplicity of infection = 3), neither BFA incubation nor COP depletion altered GP64 surface display rate or low-pH-induced cell-cell fusion. Quantitatively, cELISA and fusion index analyses (4) confirmed comparable surface/total ratios and fusogenicity between treated and control BmN samples (Fig. 1B). qPCR analysis confirmed that siRNA targeting COPI and COPII effectively knocked down the expression of the corresponding subunits (Fig. 1C). In contrast, BFA treatment strongly inhibited the surface localization of vesicular stomatitis virus glycoprotein G (VSV-G), a prototypical Golgi-dependent viral glycoprotein, in BmN cells (Fig. 1D), confirming that the ER–Golgi pathway was functionally disrupted under the same experimental conditions.

**TABLE 1:** Target sequence of siRNA and primers

Name	Sequence (5′→3′)
Target sequences for RNAi	
SiCOPI (Gene ID: 100379186, *B. mori*)	GGAGATGACTACAAGATTA
SiCOPII (Gene ID: 101743064, *B. mori*)	AGTTCATCCAGCAGAATGA
SiCOPI (Gene ID: 118270806, *S. frugiperda*)	CCTACAAGCTTGCGAGAAAT
SiCOPII (Gene ID: 118275263, *S. frugiperda*)	AGAAATACATGCAGAGCAAT
Primers for qPCR	
COPIQF (Gene ID: 100379186, *B. mori*)	CAGCAACTGTACATGCAGGC
COPIQR (Gene ID: 100379186, *B. mori*)	CTGCAGCAGATGCGATCAAT
COPIIQF (Gene ID: 101743064, *B. mori*)	GCCGATACCATCTGCGGAC
COPIIQR (Gene ID: 101743064, *B. mori*)	CAGTCGAAGAGACGGCCAA
COPIQF (Gene ID: 118270806, *S. frugiperda*)	CCTATTTGGACAGGCGGACG
COPIQR (Gene ID: 118270806, *S. frugiperda*)	AGAGTTTGACCTGTCGGTCG
COPIIQF (Gene ID: 18275263, *S. frugiperda*)	GCTCACAGGCCCGGTT
COPIIQR (Gene ID: 18275263, *S. frugiperda*)	TCGCAGAATTAGAAAGCCAACA
GAPDH QF	CATTCCGCGTCCCTGTTGCTAAT
GAPDH QR	GCTGCCTCCTTGACCTTTTGC
Primers for pIZ/V5-VSVG cloning	
VSV-GF (*Bam*HI)	TCAGGATCCATGAAGTGCCTTTTGTAC
VSV-GR (*Xho*I)	ACTCTCGAGTTACTTTCCAAGTCGGTT

**Fig 2:**
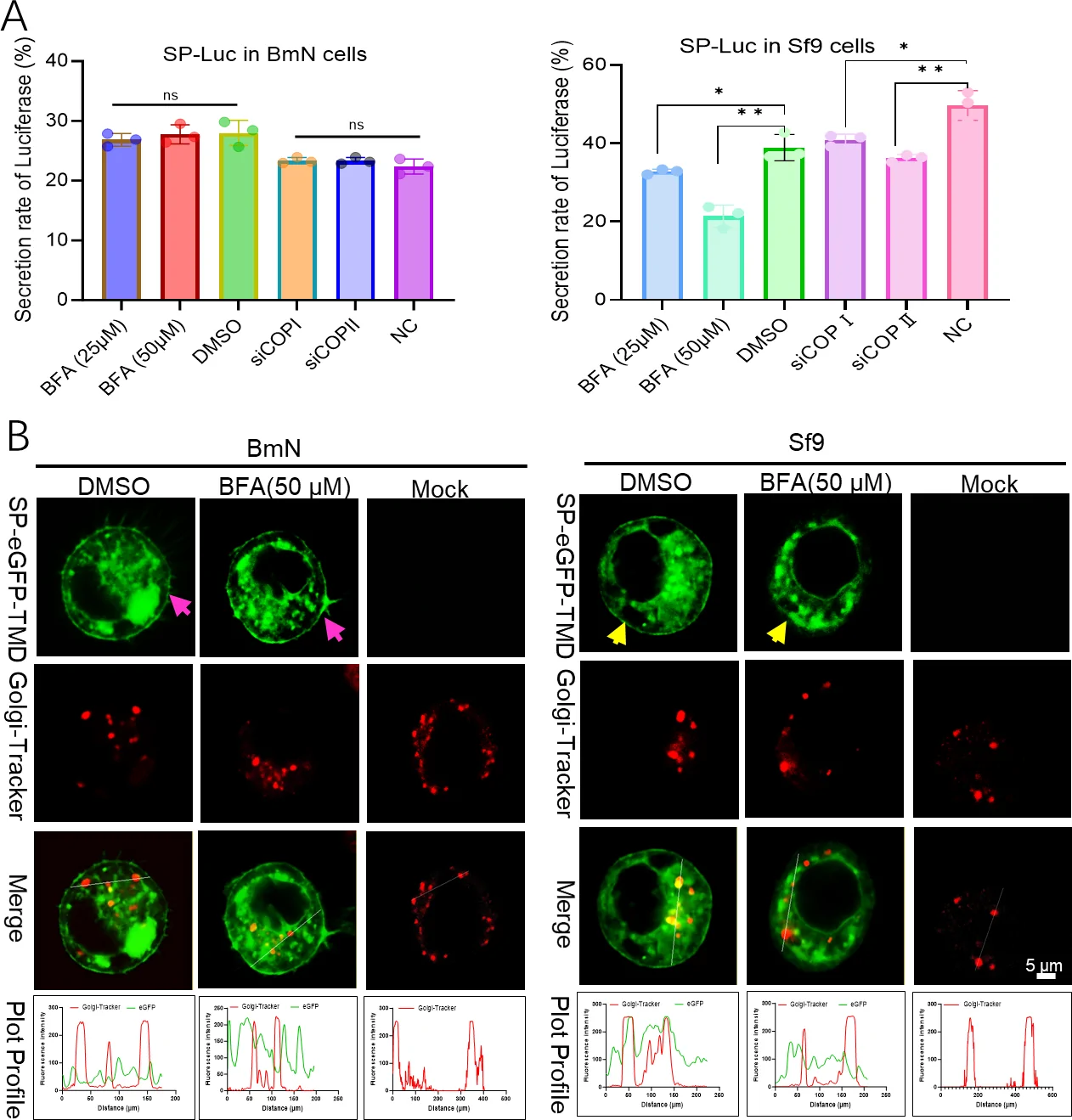
The signal peptide mediates Golgi-independent trafficking in BmN but not Sf9 cells. (**A**) Secretion of SP-guided luciferase (SP-Luc) under ER–Golgi disruption. Stable BmN and Sf9 cells expressing SP-Luc were constructed as described (7) and were either treated with BFA for 4 h or subjected to siRNA-mediated depletion of COP complex subunits. SP-Luc secretion was quantified by luciferase assay and presented as mean ± SD (*n* = 3). Statistical significance is indicated (*P < 0.05, **P < 0.01; ns, not significant). (**B**) Subcellular localization of SP-eGFP-TMD under ER-Golgi disruption. BmN and Sf9 cells were transfected with SP-eGFP-TMD as described (5) and treated with DMSO or BFA for 4 h, followed by confocal microscopy analysis using a Golgi marker at 72 h p.t. Line-scan fluorescence intensity profiles (dashed lines) were generated using ImageJ. Green, SP-eGFP-TMD; red, Golgi marker. Pink arrows indicate PM localization in BmN cells; yellow arrows indicate PM localization in Sf9 cells; white arrows indicate colocalization with the Golgi. The Mock control shows Golgi staining alone. Scale bar, 5 μm.

We next examined whether the SP alone is sufficient to drive direct Golgi-independent trafficking. BmNPV GP64 SP (36 residues) was fused to luciferase (SP-Luc) to generate a transient expression vector and was expressed in cells as described (7). SP-Luc secretion was insensitive to ER-Golgi inhibition in BmN cells but was strongly inhibited in Sf9 cells (Fig. 2A). Likewise, SP-eGFP-TMD (7) localized to the PM in BmN cells under BFA treatment, whereas PM targeting was blocked in Sf9 cells (Fig. 2B). Confocal microscopy combined with line-scan analysis revealed minimal colocalization between GP64 and Golgi markers in BmN cells, consistent with a trafficking route independent of the Golgi apparatus. In addition, depletion of six host factors (8) involved in Golgi-to-PM trafficking did not affect GP64 surface expression (data not shown), further indicating that GP64 trafficking does not rely on the conventional Golgi-to-PM machinery. Notably, SP retention also reshaped the glycosylation profile of GP64 compared with AcMNPV GP64 (6), suggesting that SP retention influences both trafficking and post-translational processing. Taken together, these results support the notion that SP-guided proteins, including GP64, can reach the PM through a Golgi-bypassing pathway in *B. mori* cells.

While our findings support a Golgi-bypassing route for GP64, several limitations should be acknowledged. The evidence for Golgi-independent trafficking is primarily indirect, relying on pathway perturbations rather than direct visualization. Although BFA can show variable efficacy in insect cells, validation with VSV-G and orthogonal COP knockdown mitigates this concern. Similarly, while residual COP protein could persist after mRNA depletion, any such residual effect would only potentially dampen the observed phenotype; while this does not invalidate our main conclusion, it should be considered as a limitation of the RNAi approach. Direct trafficking analyses and cleaner viral output measurements will be pursued in future studies. Despite these limitations, multiple independent lines of evidence consistently support a Golgi-bypassing route for GP64 in *B. mori* cells.

In summary, our results show that BmNPV GP64 is delivered to the cell surface via a Golgi-independent secretory route that is associated with SP retention. This host-specific trafficking mechanism expands current understanding of viral glycoprotein transport and suggests that SP retention may represent an adaptive strategy in baculovirus–host interactions.

## Data Availability

The data that support the findings of this study are available from the corresponding author upon reasonable request.
